# Label-free detection of brain tumors in a 9L gliosarcoma rat model using stimulated Raman scattering-spectroscopic optical coherence tomography

**DOI:** 10.1117/1.JBO.26.7.076004

**Published:** 2021-07-14

**Authors:** Soheil Soltani, Zhe Guang, Zhaobin Zhang, Jeffrey J. Olson, Francisco E. Robles

**Affiliations:** a Georgia Institute of Technology and Emory University, Wallace H. Coulter Department of Biomedical Engineering, Atlanta, Georgia, United States; b Emory University, Winship Cancer Institute, Atlanta, Georgia, United States; c Emory University School of Medicine, Department of Neurosurgery, Atlanta, Georgia, United States

**Keywords:** optical coherence tomography, stimulated Raman scattering, molecular imaging

## Abstract

**Significance:** In neurosurgery, it is essential to differentiate between tumor and healthy brain regions to maximize tumor resection while minimizing damage to vital healthy brain tissue. However, conventional intraoperative imaging tools used to guide neurosurgery are often unable to distinguish tumor margins, particularly in infiltrative tumor regions and low-grade gliomas.

**Aim**: The aim of this work is to assess the feasibility of a label-free molecular imaging tool called stimulated Raman scattering-spectroscopic optical coherence tomography (SRS-SOCT) to differentiate between healthy brain tissue and tumor based on (1) structural biomarkers derived from the decay rate of signals as a function of depth and (2) molecular biomarkers based on relative differences in lipid and protein composition extracted from the SRS signals.

**Approach**: SRS-SOCT combines the molecular sensitivity of SRS (based on vibrational spectroscopy) with the spatial and spectral multiplexing capabilities of SOCT to enable fast, spatially and spectrally resolved molecular imaging. SRS-SOCT is applied to image a 9L gliosarcoma rat tumor model, a well-characterized model that recapitulates human high-grade gliomas, including high proliferative capability, high vascularization, and infiltration at the margin. Structural and biochemical signatures acquired from SRS-SOCT are extracted to identify healthy and tumor tissues.

**Results**: Data show that SRS-SOCT provides light-scattering-based signatures that correlate with the presence of tumors, similar to conventional OCT. Further, nonlinear phase changes from the SRS interaction, as measured with SRS-SOCT, provide an additional measure to clearly separate tumor tissue from healthy brain regions. We also show that the nonlinear phase signals in SRS-SOCT provide a signal-to-noise advantage over the nonlinear amplitude signals for identifying tumors.

**Conclusions**: SRS-SOCT can distinguish both spatial and spectral features that identify tumor regions in the 9L gliosarcoma rat model. This tool provides fast, label-free, nondestructive, and spatially resolved molecular information that, with future development, can potentially assist in identifying tumor margins in neurosurgery.

## 1 Introduction

Resection is the first line therapy to manage brain tumors, where the survival rate of patients increases with the extent of resection.^1^^–^^3^ However, it is also critically important to minimize removal of healthy brain tissue to avoid deficits in brain function, which could have severe consequences for patients. Thus the ability to distinguish brain tumor from healthy tissue is paramount in neurosurgery, as it directly relates to both patients’ overall survival and quality of life.^4^^–^^6^ Even though there are pathological and biochemical differences between tumor and benign tissues, identifying tumor margins intraoperatively remains a significant challenge.

Various imaging techniques have been proposed to help guide neurosurgery, including magnetic resonance imaging,^7^^,^^8^ computed tomography,^9^ 5-aminolevulinic acid fluorescence,^10^ and ultrasound.^11^ However, these methods have important limitations, such as very high costs, lack of *in vivo* imaging capabilities, and lack of sensitivity for infiltrative tumors and low-grade disease. More recently, label-free optical methods have been proposed; these offer important benefits, including low-cost, high-resolution, and potentially highly specific *in vivo* tumor margin detection. Optical coherence tomography (OCT), for instance, has been used to guide brain cancer surgery by leveraging the optical attenuation difference between tumor and benign tissues.^12^ Another example is stimulated Raman scattering (SRS), which measures the intrinsic vibrational properties of molecules.^13^ Previous studies have shown that lipid and protein contents, as measured with SRS, can differentiate between healthy and tumor brain tissues.^14^^–^^16^ SRS has indeed been successfully applied to guide neurosurgery but only using excised tissues,^17^^–^^21^ and its implementation for *in vivo* intraoperative image guidance has been challenging.^17^^,^^22^^–^^24^ Further, unlike OCT, SRS is a point-scanning method and does not offer the convenient spatial multiplexing capability that renders OCT a fast volumetric imaging tool. Nevertheless, the unique molecular information available with SRS—particularly from proteins and lipids, which have a high Raman cross section—makes it an attractive candidate to identify tumor margins based on biochemical contrast.

In this work, we apply a method, called stimulated Raman scattering-spectroscopic optical coherence tomography (SRS-SOCT),^25^^,^^26^ and assess its potential to identify tumor margins for image-guided neurosurgery. SRS-SOCT combines the spatial and spectral multiplexing capabilities of SOCT with the molecular sensitivity and specificity of SRS to provide fast, label-free, tomographic molecular information, thus overcoming the limitations of each individual technique (OCT and SRS). In this work, we utilize a 9L gliosarcoma rat tumor model—a well-characterized model of human high-grade gliomas, that expresses high proliferative capability, high vascularization, and infiltration at the margin^27^^,^^28^—to assess the potential of SRS-SOT to help guide neurosurgery. Results demonstrate that SRS-SOCT can distinguish both spatial and spectral features that identify tumor regions. This unique method may become an important tool for brain tumor detection.

## 2 Methods and Materials

### 2.1 Nonlinear Optical SRS Process

**Fig. 1:**
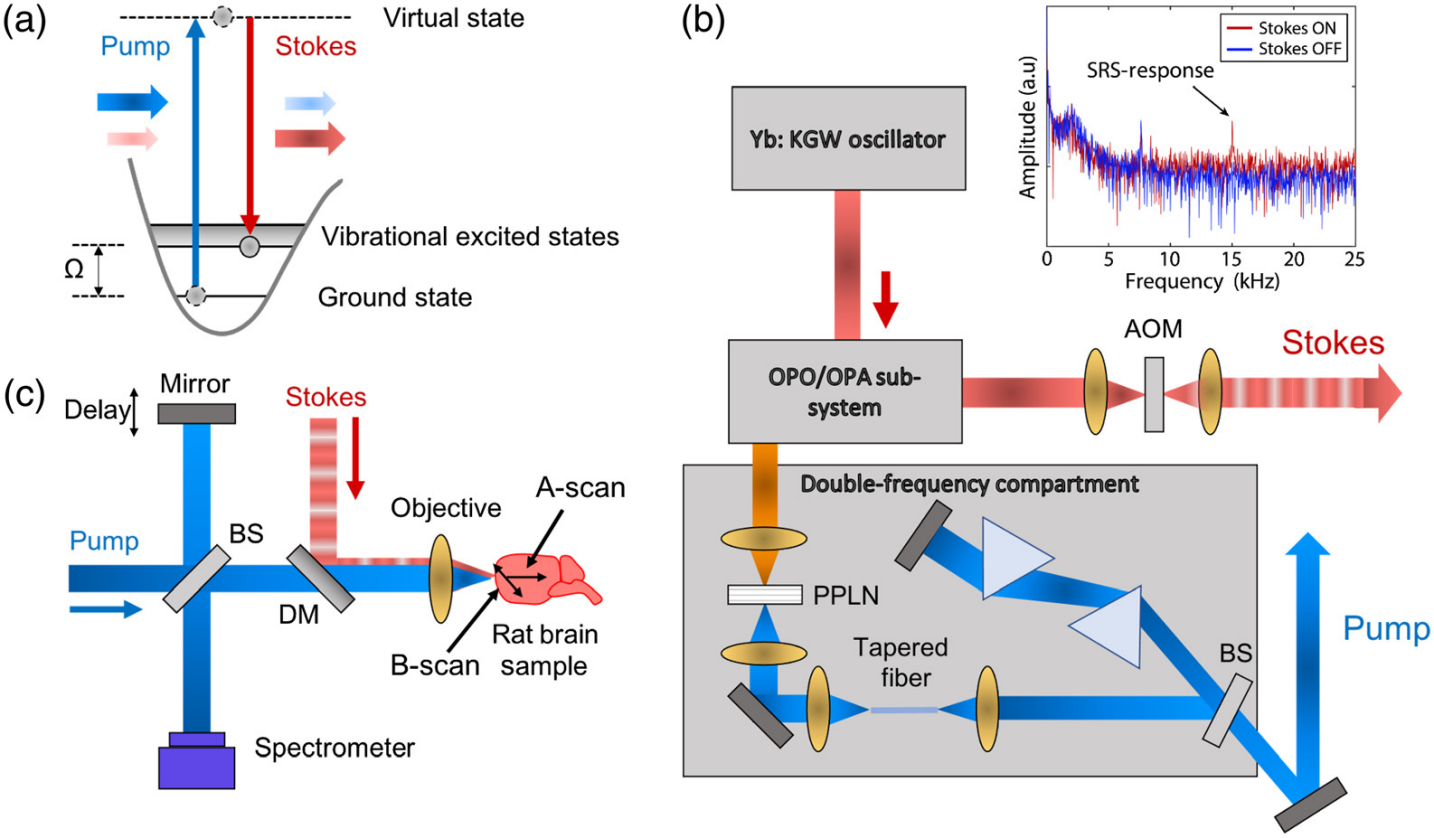
Schematic of SRS interaction and experimental SRS-SOCT setup. (a) SRS process energy diagram. (b) An ultrafast laser system designed to deliver pump and Stokes beams for SRS-SOCT. (c) The imaging setup, with a spectroscopic detection using a Michelson interferometer. DM, dichroic mirror; BS, beam splitter; OPO, optical parametric oscillator; OPA, optical parametric amplifier; AOM, acoustic optical modulator; and PPLN, periodically poled lithium niobate. In the inset of (b), we show measured signals in the frequency domain, with the SRS-response peak at 15 kHz, given by the AOM modulation frequency. The peak only appears when Stokes beam is on, indicating the nonlinear optical process.

SRS occurs when there is an energy transfer from a high-energy pump photon to a low-energy Stokes photon via vibrational interactions with a molecule, as shown in Fig. 1(a). In this case, the pump beam excites a biomolecule from its ground state to a virtual state. Then when the Stokes beam (instantaneously) stimulates the molecule back down to a vibrational state, a coherent Stokes photon is generated. The frequency difference between the pump and Stokes beams is denoted as $\Omega =\omega_{\text{pump}}-\omega$. The nonlinear change experienced by the pump field is expressed as

$$E(\omega )\propto E_{0}(\omega )\exp(-i\frac{{\overset{˜}{n}}_{\mathrm{NL}}(\omega )\omega \cdot 2z_{0}}{c}),$$ (1)

$${\overset{˜}{n}}_{\mathrm{NL}}(\omega )=\frac{3}{4n_{0}^{2}\varepsilon_{0}c}\chi^{\left(3\right)}(\Omega )I_{\text{Stokes}},$$ (2)

where $E_{0}(\omega )$ is the initial incident pump field, ${\overset{˜}{n}}_{\mathrm{NL}}(\omega )$ is the complex nonlinear refractive index of the medium, $z_{0}$ is the interaction length, $I_{\text{Stokes}}$ is the Stokes beam intensity, $n_{0}$ is the refractive index of the medium, and $\varepsilon_{0}$ is the permittivity of free space. Note that the complex properties of ${\overset{˜}{n}}_{\mathrm{NL}}(\omega )$ can be directly measured with SRS-SOCT and are proportional to the third-order nonlinear optical susceptibility $\chi^{\left(3\right)}$, which yields highly specific biochemical (Raman) signatures. As discussed below in Sec. 2.3, here we target the high-wavenumber region of the Raman spectrum, which (1) provides molecular information from lipids and proteins and (2) has been used to identify brain tumors.^18^

### 2.2 Ultrafast Laser System for SRS-SOCT

We recently introduced an ultrafast laser system specially designed for SRS-SOCT.^26^ The schematic of the system is shown in Fig. 1(b). Overall, this system operates at a 40-MHz repetition rate with two outputs: one for the Stokes beam and the other for the pump beam, which also serves at the SOCT light source, as illustrated in Fig. 1.

For the SRS pump pulse, first, a high-power (∼8  W) Yb:KGW oscillator (∼450  fs, 1040 nm) is used as the pump source for the laser system. The output is then sent to an optical parametric oscillator (OPO) and an optical parametric amplifier (OPA) to produce a tunable output from 1.4 to 2  μm. Then the pulse is frequency doubled using a periodically poled lithium niobate (PPLN) crystal, which produces a narrow band pulse at 790 nm. Next, the pulse is spectrally broadened via self-phase modulation using a tapered SMF28 fiber (78-mm length). This step is critical to generating a low noise, compressible broadband pulse that is well suited for SRS and SOCT. Finally, a prism pulse compressor is used to compensate for chromatic dispersion and shorten the duration of the output pulse (∼50  fs).

For the SRS Stokes pulse, we directly split a portion of the Yb:KGW oscillator’s output. This pulse is also actively modulated at 15 kHz by an acoustic optical modulator (AOM, Gooch & Housego, AOMO 3200-124) for lock-in detection.

Overall, our laser system outputs a pump pulse centered at 790 nm (but tunable from 740 to 975 nm) with a bandwidth of ∼40  nm and compressible duration down to <50  fs. The average pump output power from the laser is set to ∼115  mW, of which <50  mW is incident on the sample. The Stokes pulse is fixed at 1040 nm, with 450 fs duration and an average output power of ∼200  mW (∼95  mW is incident on the sample). As with other studies,^13^^,^^17^^,^^18^ no noticeable tissue damage was produced at these power levels. With this configuration, we achieve an axial resolution with OCT of ∼10  μm and cover a Raman spectral region of ∼2700 to $3200\;{\mathrm{cm}}^{-1}$. The SRS signal shares the same axial resolution as OCT, but as with any SOCT method, the molecular signatures accumulate along depth.^25^^,^^26^ The SRS-SOCT signal scaling with pump and Stokes power has been rigorously characterized in our prior work using well controlled synthetic samples and fresh tissues.^25^^,^^26^

### 2.3 Interferometric Spectroscopic Detection in SRS-SOCT

The imaging portion of the SRS-SOCT system is shown in Fig. 1(c). First, the SRS pump beam output from the laser is sent to a Michelson interferometer where the light is split into two identical beams: one used as the reference beam and the other used as the sample beam. The Stokes beam, modulated at 15 kHz by an AOM as described above, is combined with the pump beam through a dichroic mirror. Both the pump and Stokes beams are focused onto the biological sample (rat brain, details below in Sec. 2.4) using a 5× objective (Zeiss, A-Plan 5×, NA = 0.12). With a beam diameter of the laser of 2 mm, this yields a lateral resolution of 5.2  μm, which applies to both the OCT and SRS signals. The generated SRS signal, encoded in the sample beam, is collected after light is backscattered and detected interferometrically as in conventional Fourier-domain SOCT. Here we use a fast spectrometer (BaySpec, OCTS-0780-0840-0900-SLIT) to record the interferometric signal. Spectra are recorded at 50 kHz (camera line scan rate), and for each A-scan, 2048 spectra are recorded. The 15-kHz Stokes beam modulation frequency is selected to be well above the thermal bandwidth of the sample to avoid any thermal contribution to the SRS signal^29^^–^^31^ {Choi, 2001 #55}. We also use a 2D Galvo-System (Thorlabs, GVS012) to steer the beams and perform lateral scans.

The recorded spectral interference between the back reflected sample field and the reference field $E_{0}(\omega )$ (same as the initial pump field) is expressed as $I_{\mathrm{spec}}(\omega )={\left|E_{0}(\omega )+rE(\omega )\exp(-i\frac{\omega \cdot 2z}{c})\right|}^{2}$, where E(ω) is given by Eq. (1), r is reflectivity, and z is the pathlength difference between the two beams (i.e., depth). From $I_{\mathrm{spec}}(\omega )$, the term of interest is the oscillating term that contains the nonlinear refractive index ${\tilde{n}}_{\mathrm{NL}}$, embedded in E(ω). Considering Eqs. (1) and (2), we express the oscillating term as

I˜spec(ω)∝rI0(ω)exp(−i2ωzc)exp(z0βω2c Im{χ(3)(Ω)}IStokes)×exp(−iz0βωc Re{χ(3)(Ω)}IStokes),   (3)

where $\beta =\frac{3}{2n_{0}^{2}c\varepsilon_{0}}$ is a constant and $I_{0}(\omega )$ is the intensity of the initial pump field (also the reference field). The second exponential term corresponds to an intensity change imposed by the imaginary part of nonlinear response $\chi^{\left(3\right)}$, and the third term corresponds to a phase change produced by the real part of $\chi^{\left(3\right)}$. Thus the amplitude and phase changes experienced by the detected signal, ΔI(Ω) and Δϕ(Ω), respectively, are given by

ΔI(Ω)∝rI0×IStokes×Im{χ(3)(Ω)}, (4)

Δϕ(Ω)∝IStokes×Re{χ(3)(Ω)}. (5)

These expressions assume a weak nonlinear perturbation, which is a reasonable approximation for SRS-SOCT.^25^^,^^32^ It is worth noting that the SRS intensity (the conventional SRS measurement) shows a dependence on the initial intensity of the pump field $I_{0}$ and sample reflectivity r, whereas the SRS phase does not. This results in the phase signal being more robust to laser power fluctuations and independent of the samples’ reflectivity,^32^ thus providing a better signal-to-noise ratio.

### 2.4 Biological Sample Preparation

**Fig. 2:**
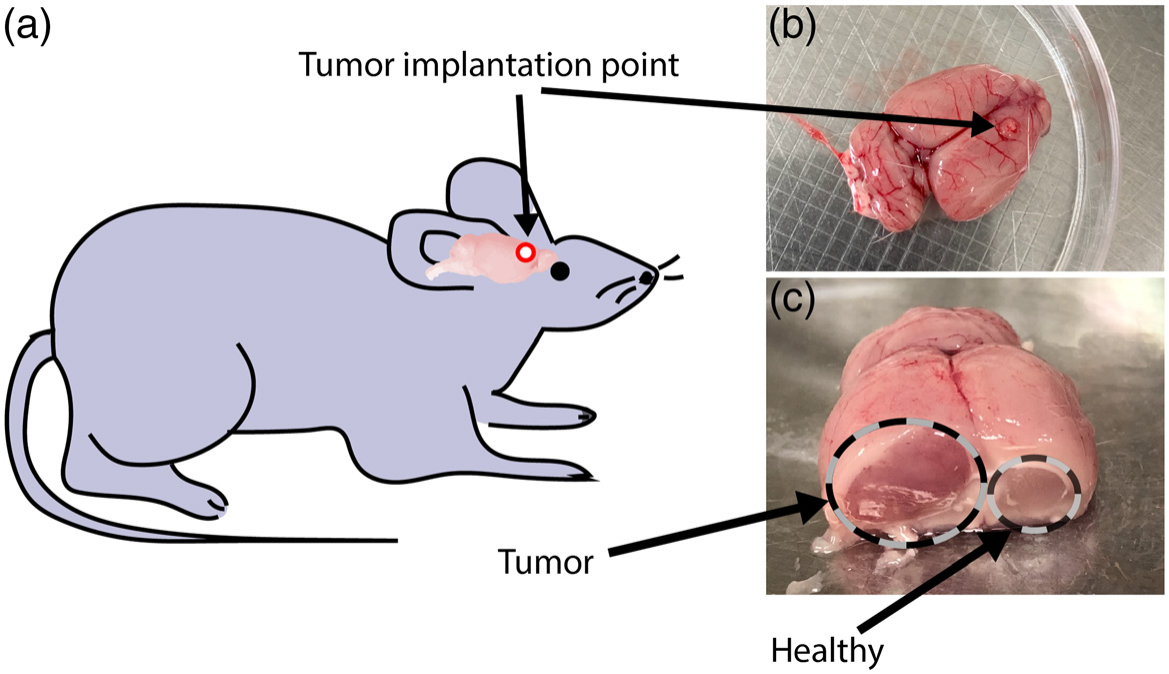
(a) Schematic of rat brain implantation. (b) Top view of the harvested brain. The implant point is indicated by the black arrow. (c) Cross section of the brain with tumor region (marked in dashed circle).

To test the ability of SRS-SOCT to detect brain tumors, we use the 9L gliosarcoma rat tumor model, which mimics human high-grade gliomas, including high proliferative capability, vascularization, and infiltrative pattern.^28^ All animal experimental protocols were approved by the Institutional Animal Care and Use Committee of Georgia Institute of Technology and Emory University. As shown in Fig. 2(a), cloned 9L glioblastoma cancer cells were implanted in N=6 Fischer rat brains. Tumor was allowed to develop for 9 to 12 days before the rats were sacrificed. To gain access to the tumor cross section, the surgically removed brains were coronally cut in two halves across the approximate tumor implantation point. SRS-SOCT imaging was performed on the tumor (identified via visual inspection) as well as the counter-lateral, healthy brain region, which was used as control. A total of 54 regions were imaged (34 from tumor regions and 20 from healthy regions). We acquired 8 to 10 scans from each brain (∼4 to 5 points from tumor regions and ∼4 to 5 points from healthy regions). Figs. 2(b) and 2(c) show photographs of a representative harvested and cut rat brain.

### 2.5 Measuring SRS-SOCT Signal

Using the SRS-SOCT system, we measured interferometric signals that contain the SRS response of rat brains. For each of the freshly cut brains (n=6), we took 8 to 10 B-scans (total of 54 B-scans), each composed of 200 A-scans (synthesized from 2048 sequential recorded spectra) along a ∼480-μm line shift. In the axial direction, the images cover a range of ∼2  mm, but the penetration into the brain tissue is only ∼0.7 to 1 mm. The scanned points were selected at equidistant positions to cover both tumor in one side and healthy regions in the counter lateral side. No staining or other sample preparations were performed for SRS-SOCT imaging. For this feasibility study (and given the use of an established animal model), all measurements were taken as independent even if they were from the same animal. Tumor regions from adjacent regions were analyzed with histopathology (using H&E stained tissue sections), and the presence of tumors was confirmed in all imaged animals. Healthy regions were also confirmed.

## 3 Results and Discussions

### 3.1 Processing SRS-SOCT Dataset

To extract the complex nonlinear SRS signals, the interferometric spectra first are interpolated from wavelength to a linear wavenumber array (k=2π/λ) and then Fourier transformed from the spectral to spatial domain to get depth information (A-scan). For each lateral position in the B-sacn, we have 2048 scans that were recorded at 50 kHz. Next, to extract the SRS response of the sample, we perform another Fourier transform along the temporal dimension to get the frequency response of the A-scans. We select only the signal corresponding to the 15-kHz modulation (i.e., lock-in detection), as shown in the inset of Fig. 1(c). This way, we obtain both the SRS amplitude and phase terms, which are then normalized by their respective background noise, as was done in Refs. 25 and 32. Finally, 200 acquisitions are synthesized to provide cross-sectional images (B-scan).

**Fig. 3:**
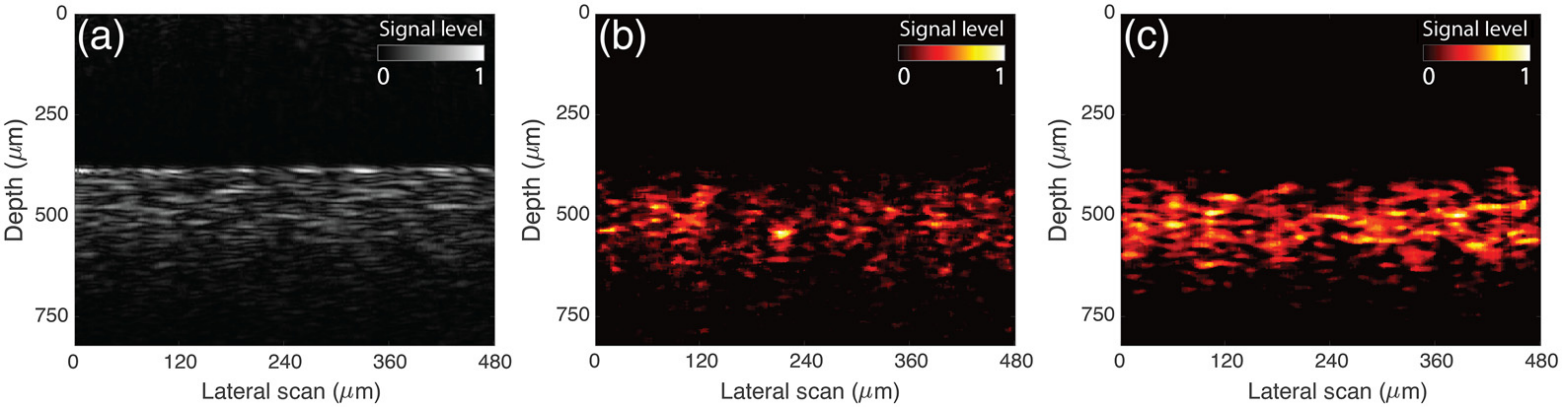
SRS-SOCT spatially resolved images (B-scans) of rat brain: (a) convectional OCT image, (b) SRS amplitude image, and (c) SRS phase image of a tumor region in rat brain. The B-scan images are synthesized from 200 A-scans (x dimension), which covers 480  μm.

An example of the SRS-SOCT B-mode image (x−z dimension) is shown in Fig. 3. Figure 3(a) shows the conventional OCT image, and Figs. 3(b) and 3(c) show SRS amplitude and phase images (average response across the high-wavenumber region). Again, the OCT image exhibits spatially resolved information from linear photon scattering, whereas the SRS-SOCT images contain spatially resolved information regarding the third-order nonlinear response of the sample.

The SRS-SOCT dataset contains both spatial and spectral information. In the following sections, we first analyze the SRS-SOCT data spectrally to give molecular specific information, and then we analyze the attenuation behavior as a function of depth from the OCT and SRS signals to show additional biomarkers that differentiate between tumor and healthy tissues.

### 3.2 Spectral Analysis of SRS-SOCT Data

As previously reported,^18^^,^^33^ the lipid and protein content of the brain tends to be lipid-rich for white matter, protein-rich for tumor regions, and lipid-and-protein-rich for cortex. These subtle molecular differences result in slight differences in the Raman spectral response, with most spectral differences arising in two particular regions in the high-wavenumber region.^18^^,^^33^ The first region is attributed to spectral signatures of lipids and roughly covers the range between 2800 and $2900\;{\mathrm{cm}}^{-1}$. The second window signifies spectral signatures of proteins and covers the regions between 2900 and $3000\;{\mathrm{cm}}^{-1}$. Thus to provide molecular specific information that yields contrast for tumors, we considered two spectral regions: a high-frequency Raman band (centered at $2947\;{\mathrm{cm}}^{-1}$) to detect proteins and a low-frequency Raman band (centered at $2861\;{\mathrm{cm}}^{-1}$) to detect lipids. These two windows provide an average cumulative SRS response of the lipid and protein contents of the sample. In this analysis, the interferometric SOCT data are filtered using a Butterworth filter with an $80\text{-}{\mathrm{cm}}^{-1}$ (∼5  nm) bandwidth at the corresponding spectral regions, and then the nonlinear amplitude and phase changes are calculated following the procedure described above in Sec. 3.1. We also note that using two windows, instead of a full time-frequency-analysis with higher spectral resolution, we achieve higher signal-to-noise ratio data, improved spatial resolution, and faster, less computationally intensive processing.

To differentiate between tumor and healthy regions, we define the following metrics: For the SRS amplitude, we chose the ratio of the high-frequency band value to the low-freuqency band value. For the SRS phase, we simply take the difference of phase values from those two bands. Thus for both of these metrics, we expect protein-rich tumor regions to have a higher amplitude ratio and higher phase difference than the normal regions. Moreover, because the SRS-SOCT signal is cummulative along depth,^25^ we extract these metrics from a depth range 100 to 150  μm from the brain surface, which allows for sufficient signal accumulation and avoids noisy, low-amplitude signals at deeper depths. Signals are averaged across each B-scan (54 regions in total) for all 6 rat brains.

**Fig. 4:**
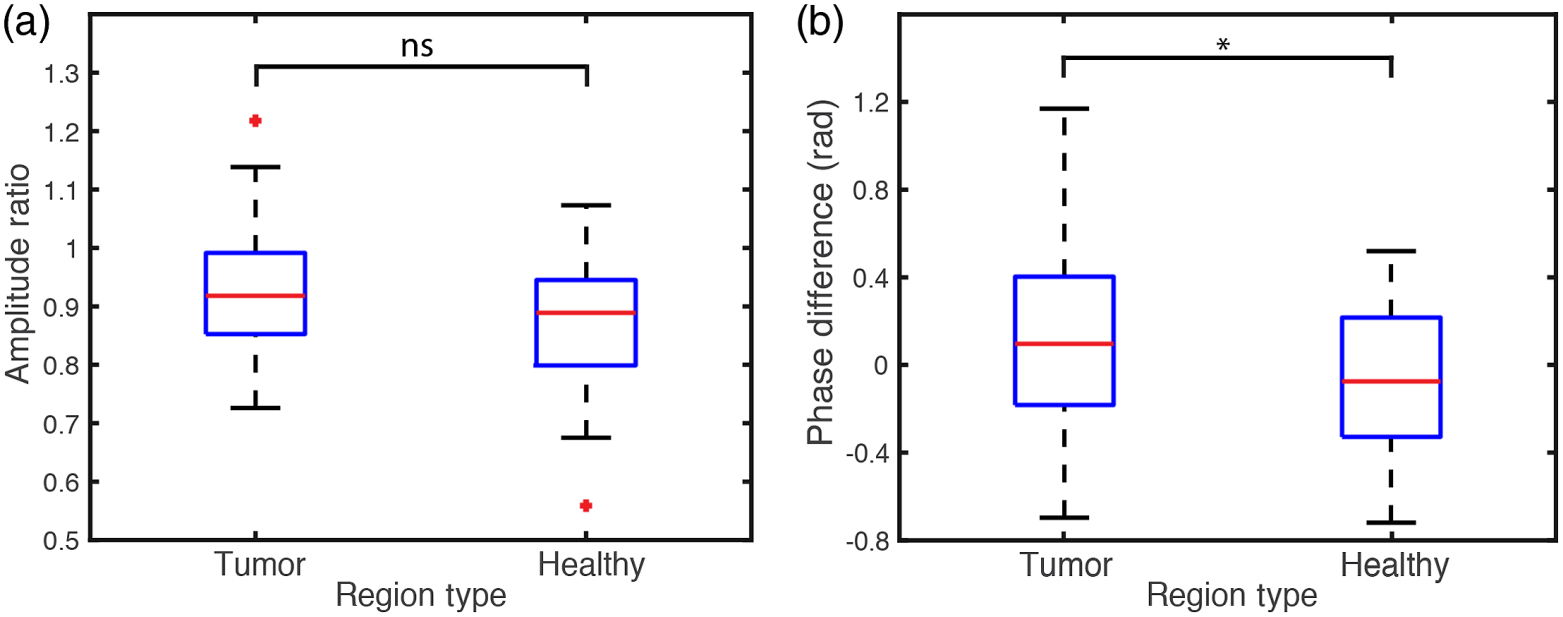
(a) Ratio of SRS amplitude of the high-frequency band value to the low-frequency band value. Differences are not statistically significant (n.s.) with a p value of 0.0973 (two-tailed t test). (b) Difference of SRS phases between the same frequency bands. p value is 0.0243 (two-tailed t test), which is statistically significant (p value<0.05), denoted by the star (*).

Results from this analysis are presented in Fig. 4 in the form of standard box plots with interquartile ranges inside the box and the whiskers indicating the nonoutlier maximum and minimum ranges. Figure 4(a) shows the SRS amplitude ratio, which shows a slightly higher amplitude ratio for tumor than healthy regions, but the difference is not statisitically significant. Figure 4(b) also shows a higher SRS phase difference for tumor regions with a p value<0.05 using a two-sided t test. As previously discussed, these results agree with our expectation that tumor regions, with a relatively rich protein composition, will have a higher phase difference, but indeed the differences are weak. Higher amplitude ratios were also expected, and while slight differences are observed, which are in agreement with these expectations, these are not statistically significant.

The spectral analysis provides insights on the local biomolecular contents, but we note that the separation between tumor and healthy regions is still small and only statistically significant for the phase signal. Further, the ability to separate between these two groups based on spectral signatures alone is very poor. These results are consistent with SRS strategies to detect tumor,^18^^,^^34^ where the spectral signatures have to be used in combination with structural parameters to achieve robust tumor detection. Thus to improve tumor identification, we incorporate structural signals from SRS-SOCT.

### 3.3 Spatial Analysis of SRS-SOCT Data

One important property of the SRS-SOCT data is the rate at which the signals attenuate as a function of imaging depth. This parameter is affected by properties of brain tissue that affect scattering and the Raman signals such as cell density, protein concentration, nuclear-to-cytoplasmic (NC) ratios, changes in myelin content, existence of foci of necrosis, and tissue heterogeneity.^12^^,^^35^^–^^39^ Further, because the SRS phase signal is contained in the argument of the interferometric signal, it is less prone to intensity variation of the pump beam and provides a more robust measurement than the nonlinear amplitude signal, and therefore it provides a higher SNR by a factor of 4 or more.^25^^,^^26^ Therefore, we only use SRS phase signal in this analysis and compare it with OCT.

**Fig. 5:**
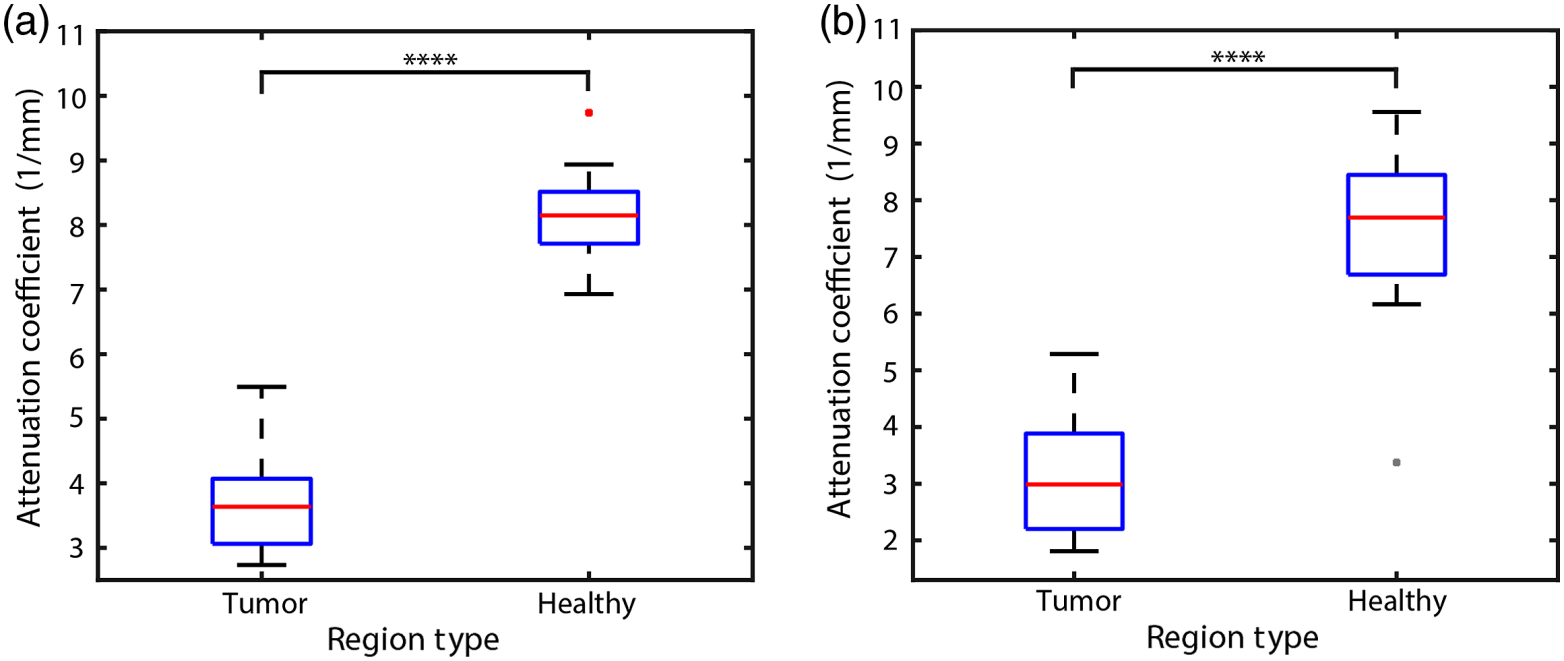
(a) Decay rate for OCT signal for rat brains. p value for tumor versus healthy is $1.74\times 10^{-16}$ (two-tailed t test). (b) Decay rate for SRS phase signal for the same brains. p value for tumor versus healthy is $3.13\times 10^{-10}$ (two-tailed t test) (n=54).

To calculate the decay rate at a point of interest, we perform an exponential fit of the OCT and SRS phase signals and calculate the average decay coefficients for each region (B-scan). To avoid artifacts generated by surface inhomogeneities, we set the starting point of the exponential fit at ∼50  μm below the sample surface. Figures 5(a) and 5(b) show plots of the decay coefficients of OCT and SRS-phase, respectively. The decay rate of healthy regions is significantly greater than tumor regions for both OCT and SRS-phase. We attribute the lower decay rate in tumor regions to changes in NC ratios as well as degradation of myelin content.^12^ Specifically, those rates associated with tumor and healthy regions in OCT signal have an average value of 3.73 and $8.17\;{\mathrm{mm}}^{-1}$, respectively, with a discriminant threshold value of $∼6\;{\mathrm{mm}}^{-1}$. Similarly, in the SRS phase signal, tumor and healthy regions exhibit average values of 3.14 and $7.83\;{\mathrm{mm}}^{-1}$, respectively, with a discriminant value of $∼6\;{\mathrm{mm}}^{-1}$. These results are in excellent agreement with the previous OCT studies that report attenuation values of 2.7 to $3.5\;{\mathrm{mm}}^{-1}$ for cancer and $7.0\;{\mathrm{mm}}^{-1}$ for healthy regions with a discriminant threshold of $∼5.5\;{\mathrm{mm}}^{-1}$.^12^^,^^40^ Our slightly higher attenuation cutoff may be attributed to the shorter center wavelength in our OCT systems (790 versus 1310 nm). The discrimination in decay rate of tumor and healthy regions suggests that OCT and SRS can both provide excellent separation between tumor and healthy tissues, with $p\text{ values}\ll 10^{-4}$.

**Fig. 6:**
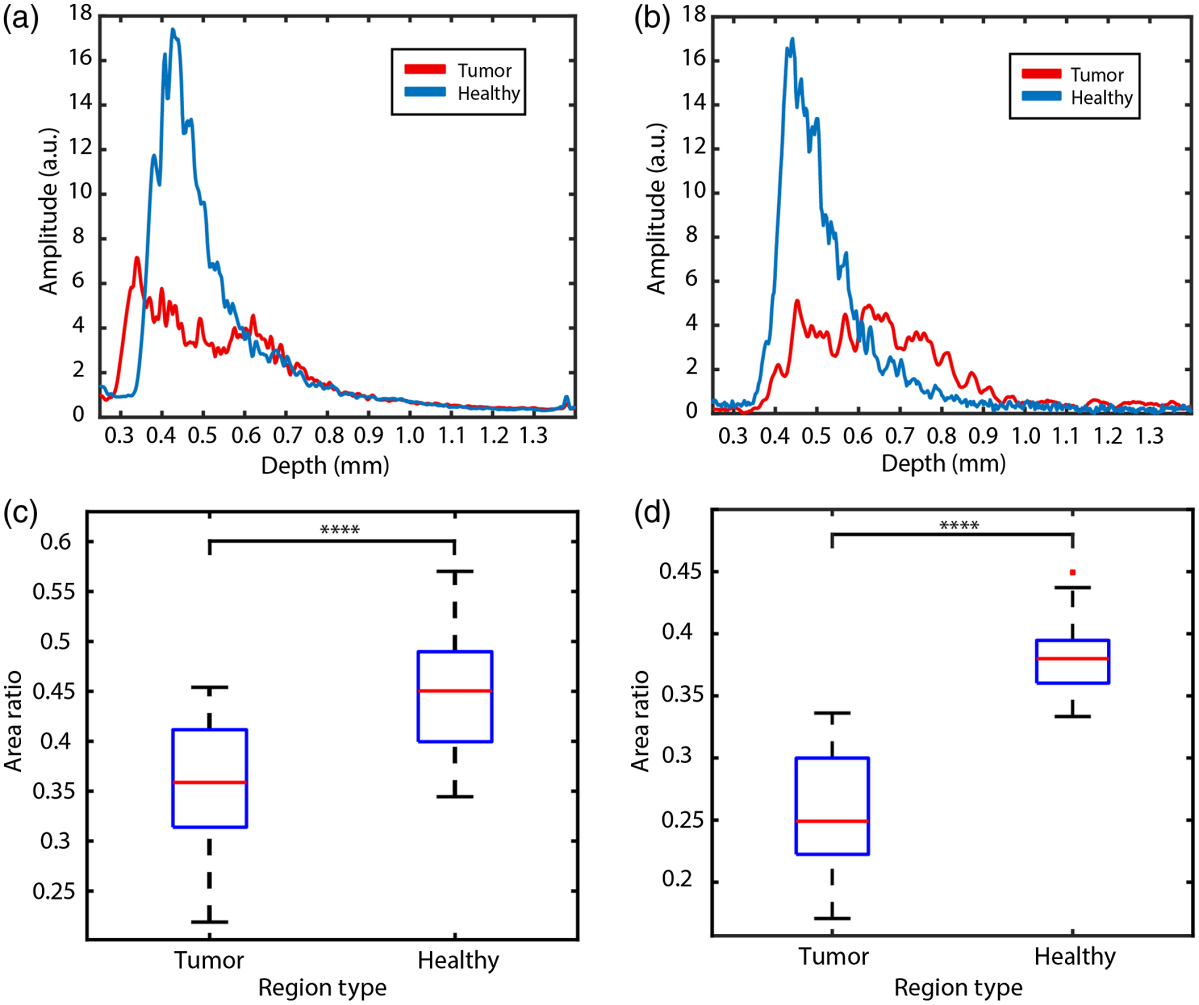
(a), (b) A-scan profiles from tumor (red) and healthy (blue) regions from (a) OCT and (b) SRS phase. (c), (d) Ratio of the area under the curve for first 100  μm from the surface to the total area under the curve, averaged across each B-scan, for (a) OCT and (b) SRS phase. p value for healthy versus tumor is $2.71\times 10^{-5}$ for OCT and (d) $1.63\times 10^{-14}$ for SRS phase. The A-scan from tumor regions show a smaller decay rate and weaker backscattering whereas healthy regions exhibit greater decay rate as well as stronger backscattering.

Finally, we note that both the OCT and SRS-phase A-scan signals in our data show a weaker backscattering response in tumor regions, despite having smaller attenuation coefficients. In other words, the A-scans of tumor regions contain weak but long-tailed signals, as opposed to the healthy regions with strong but short-tailed signals [Figs. 6(a) and 6(b)]. To show the differences in A-scans of the tumor versus healthy regions, we calculate the ratio of the area under curve for the first 100  μm from the surface, relative to the total area under the curve. As shown in Figs. 6(c) and 6(d), it is clear that the tumor regions exhibit a weaker scattering and SRS-phase response compared with healthy regions. Decreased scattering coefficient of brain tumor is attributed to several key factors such as cell density, NC ratio, and alterations in myelin content.^12^ This results in a lower decay rate in brain tumor regions.

**Fig. 7:**
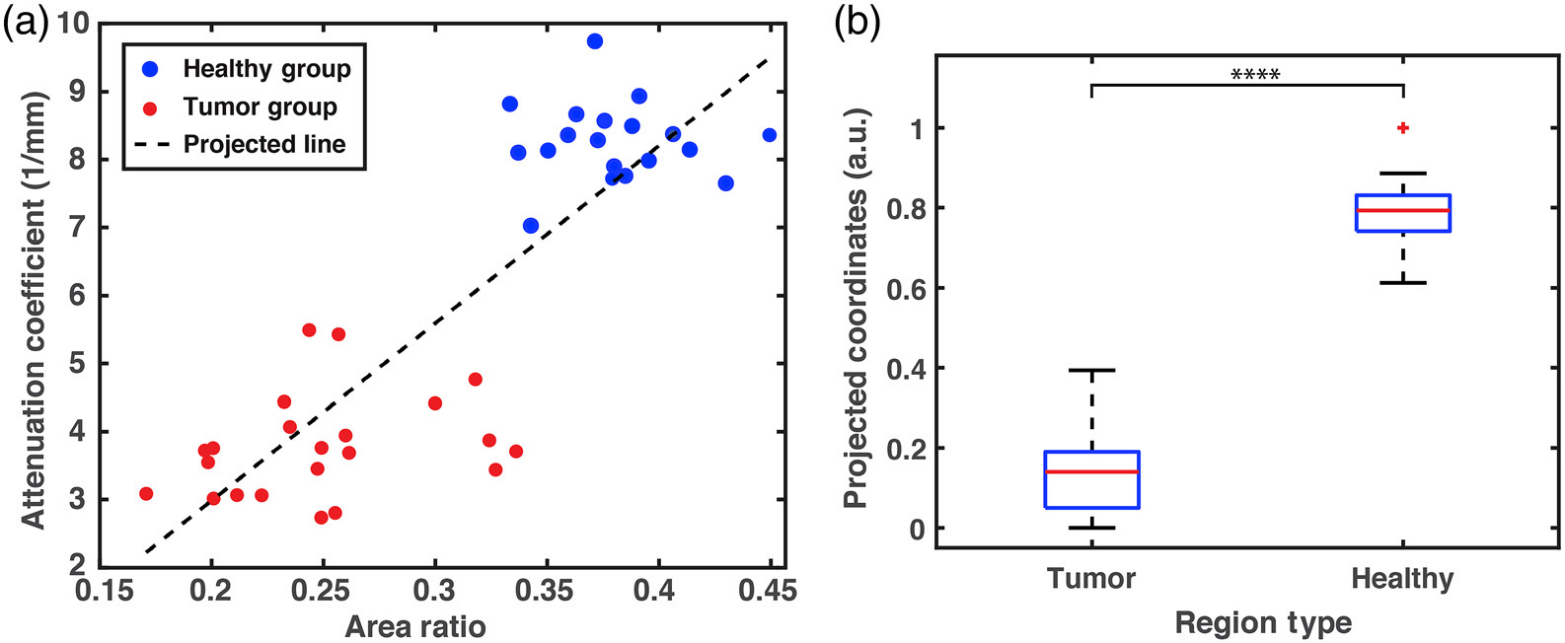
(a) Scatter plot of attenuation coefficient of OCT signal versus SRS-SOCT phase-signal area ratio. (b) The box plot of the projected distance of the scattered points from a reference point along the projection line in (a).

Although indeed the OCT and SRS-SOCT parameters show similar discrimination levels between healthy and tumor regions alone, the combination of these metrics provides complimentary information that enhances the combined method’s ability to identify tumors. To illustrate this point, Fig. 7(a) plots the attenuation coefficient of OCT versus the SRS-SOCT phase-signal area ratio, where this combined 2D parameter space provides better differentiation between tumors and healthy tissues than either of the two parameters alone. Next, we compute the coordinates of each point along the dashed line in Fig. 7(a) (rescaled from 0 to 1), which represents the direction along which the two groups are most distinguishable. Of note, the line is a diagonal, meaning that the two parameters together yield the best separation. These coordinates are then used to calculate statistical significance and generate a boxplot as shown in Fig. 7(b). Results yield a p value of $6.63\times 10^{-23}$, which is highly statistically significant.

## 4 Conclusions

As a label-free nonlinear optical method, SRS has the advantages of being nondestructive and sensitive to the biomolecular contents of biological tissues. SOCT, on the other hand, provides fast and spatially-and-spectrally resolved details of linear scattering structures but lacks molecular targets. By combining these two methods, SRS-SOCT provides rich molecular information with the spatial and spectral multiplexing capabilities of SOCT. Here we have shown that SRS-SOCT has the capability of yielding distinguishable features of brain tumor and healthy tissues in both the spectral and spatial domains, using a 9L gliosarcoma rat tumor model. In addition, we have shown that SRS-SOCT reveals features (e.g., phase area ratio, which combines Raman signals and structure) with high differentiation, whereas spectral features on their own still require further improvements on the system for higher differentiation. Multiple parameters can also be quantified from the SRS-SOCT data, which provide complimentary information and jointly yield the strongest differentiation between normal brain and tumor tissue. Moreover, the fast data acquisition provided by OCT enables spatially resolved SRS maps over relatively large regions. In our setup, an A-scan only takes a fraction of a second to complete and a B-scan with molecular information covering an area of ∼0.5  mm×1  mm (lateral X axial) is captured in a few seconds. This speed may enable timely diagnostic feedback during brain surgery based on the tumor’s structural and biochemical features.

Future work will continue to enhance the SRS signal levels. Improved management of dispersion and control of the laser power may yield a shorter and stronger pump pulse (below the damaging threshold) inside biological samples, which would improve the SRS signal level. Further, as previously demonstrated, our laser system has near-shot-noise characteristics at frequencies above 250 kHz;^26^ therefore, noise can be further reduced by operating at frequencies closer to or above 250 kHz. This may be possible using emerging fast line cameras. For this tool to progress to clinical use, one also needs to develop a convenient pulse delivery approach, using for example a hand-held device or articulated arm, that will require future development.
